# Multicenter Prospective Comparative Study of Patient Radiation Doses in Localization Techniques for Small Lung Lesions

**DOI:** 10.3390/cancers17193119

**Published:** 2025-09-25

**Authors:** Tomoki Nishida, Yuichi Saito, Takeshi Takata, Shizuka Morita, Ryo Takeyama, Shinya Kohmaru, Tomohiro Watanabe, Nobuo Yamaguchi, Hikaru Takahashi, Yasuyuki Kanamoto, Hiroaki Morooka, Takayuki Ibi, Yoshikane Yamauchi, Ryuta Fukai, Nobumasa Takahashi, Tetsu Kanauchi, Ikuo Kobayashi, Masafumi Kawamura, Yukinori Sakao

**Affiliations:** 1 Department of Surgery, Teikyo University School of Medicine, Tokyo 173-0003, Japan; nishidatomoki.322@gmail.com (T.N.); morita.shizuka.ap@teikyo-u.ac.jp (S.M.); 22md10020zd@stu.teikyo-u.ac.jp (R.T.); ahinsy@gmail.com (S.K.); basketball.58touitai@gmail.com (T.W.); takahashi.hikaru.qq@teikyo-u.ac.jp (H.T.); piercetheskywithyourdrilll@docomo.ne.jp (Y.K.); yoshikaney@med.teikyo-u.ac.jp (Y.Y.); ysakao070@gmail.com (Y.S.); 2 Shonan Kamakura General Hospital, Kamakura 247-8533, Japan; dowarfu@gmail.com (N.Y.); ryuta.f@hotmail.co.jp (R.F.); 3 Advanced Comprehensive Research Organization, Teikyo University, Tokyo 173-0003, Japan; takata@med.teikyo-u.ac.jp; 4 Saitama Cardiovascular and Respiratory Center, Kumagaya 360-0197, Japan; morooka.hiroaki80@gmail.com (H.M.); t-ibi@nms.ac.jp (T.I.); ntakahas030417@gmail.com (N.T.); kanauchi.tetsu@saitama-pho.jp (T.K.); 5 Research Institute of Nuclear Engineering, University of Fukui, Fukui 910-0017, Japan; Kobayashi.landauer@gmail.com; 6 Teikyo University Shinjuku Clinic, Tokyo 160-0022, Japan; mkawamur@med.teikyo-u.ac.jp

**Keywords:** radiation dose, cone-beam computed tomography, hook-wire, VAL-MAP, small lung cancer

## Abstract

**Background/Objectives:** Although surgeries employing cone-beam computed tomography (CBCT) for small lung lesions have been reported, the association between CBCT scan frequency and patient radiation exposure remains unclear. This study aimed to investigate patient radiation doses from CBCT during thoracic surgeries, and the patient radiation doses were compared with those from other preoperative marking methods. **Methods**: This multicenter prospective study included 81 patients who underwent surgery for small lung lesions requiring marking between January 2021 and June 2024 at three institutions. CBCT-guided surgeries involved the use of metal clips in a hybrid operating room with 1–4 scans, depending on the lesion. For other preoperative marking methods, hook-wire or virtual-assisted lung mapping (VAL-MAP) was used. Patient radiation doses were measured using wearable dosimeters at five anterior thorax sites, and the total dose was compared across methods. **Results**: The study included 81 patients: CBCT (*n* = 61), VAL-MAP (*n* = 10), and hook-wire (*n* = 10). CBCT cases were distributed as follows: single scan (*n* = 10), double scans (*n* = 34), triple scans (*n* = 15), and quadruple scans (*n* = 2). The radiation doses were 86.9 ± 61.7 mGy for hook-wire, 39.8 ± 27.5 mGy for VAL-MAP, and 11.0 ± 6.5 mGy for single-scan CBCT, 17.3 ± 7.8 mGy for double scans, 23.1 ± 14.0 mGy for triple scans, and 22.7 ± 0.1 mGy for quadruple scans. Although radiation exposure increased with more CBCT scans, performing up to triple scans resulted in significantly lower exposure compared to other methods. **Conclusions**: Intraoperative CBCT is a feasible and safe technique for identifying small lung lesions, providing lower radiation exposure compared to other preoperative localization methods.

## Simple Summary

As sublobar resections for small lung nodules become more common, various localization techniques have been developed to guide surgeons during minimally invasive procedures. We use intraoperative cone-beam computed tomography (CBCT) to localize target lesions, but its radiation exposure has not been well established. In this study, we compared radiation doses from CBCT with those from commonly used methods such as hook-wire and virtual-assisted lung mapping (VAL-MAP) at other institutions. Our results showed that CBCT, when limited to triple scans or fewer, resulted in lower radiation exposure than the other techniques. Furthermore, CBCT was feasible even in cases involving multiple lung lesions. While none of the methods reached harmful levels of radiation, our findings provide important baseline data on patient exposure during small lung cancer surgeries. This information may help refine future localization strategies as sublobar resections become increasingly precise.

## 1. Introduction

In 2022, lung cancer was the most frequently diagnosed cancer, accounting for 2.5 million new cases (12.4% of all cancers), and the leading cause of cancer-related death with an estimated 1.8 million deaths (18.7%) according to GLOBOCAN 2022 [1]. The widespread implementation of low-dose computed tomographic (LDCT) screening has led to increased detection of small pulmonary nodules, many requiring surgical intervention [2,3,4]. Recent studies have demonstrated the feasibility and safety of sublobar resections, including segmentectomy and wedge resection, for early-stage non-small cell lung cancer [5,6,7,8].

The shift toward minimally invasive surgical approaches has created a critical need for accurate localization methods, as thoracoscopic and robotic techniques do not allow direct palpation of pulmonary lesions. Securing adequate surgical margins remains paramount to prevent local recurrence, particularly challenging for small or deeply situated nodules. Various localization techniques have been developed, each with distinct advantages and potential risks.

Hook-wire localization, first reported in 1992, involves percutaneous placement of a wire anchor into the lung under computed tomography (CT) guidance [9,10]. While effective, complications including wire dislodgement, pneumothorax, bleeding, and rare but fatal air embolism have been reported [11,12,13]. To address these safety concerns, bronchoscopic approaches were developed [14,15,16] and virtual-assisted lung mapping (VAL-MAP) was introduced by Sato et al. in 2014 [17,18]. VAL-MAP uses transbronchial dye marking guided by virtual bronchoscopy, demonstrating improved safety profiles. However, its implementation requires specialized equipment and expertise, limiting widespread adoption.

Intraoperative cone-beam CT (CBCT) in hybrid operating rooms (OR) represents a newer approach, allowing real-time localization during surgery [19]. This technique potentially offers advantages, including elimination of marker dislodgement risk and the ability to target multiple lesions in a single procedure. However, a critical clinical question remains regarding radiation exposure, particularly with multiple scans required for complex cases.

Despite the growing use of these techniques, comprehensive comparative data on patient radiation exposure are lacking. As we previously reported basic data on patient radiation exposure in hybrid OR settings where CBCT was used intraoperatively during thoracic surgery [20], prior studies have described radiation doses for individual methods. However, direct comparisons using standardized measurement approaches are lacking in the literature. This knowledge gap hinders evidence-based selection of optimal localization strategies and informed patient counseling regarding radiation risks.

This multicenter prospective study aimed to directly compare patient radiation doses among three commonly used localization techniques for small lung lesions: intraoperative CBCT, VAL-MAP, and CT-guided hook-wire placement. We sought to determine whether CBCT results in higher radiation exposure compared to established methods and to establish baseline comparative data for clinical decision-making and future protocol development.

## 2. Materials and Methods

### 2.1. Study Design and Ethical Considerations

This multicenter prospective observational study was conducted at three specialized institutions between January 2021 and June 2024. The study was approved by the institutional review board of Teikyo University School of Medicine on 23 June 2020 (approval number: 20-068), serving as the central ethics committee for all participating institutions. The study was conducted in accordance with the principles of the Declaration of Helsinki, as revised in 2013. Clinical data were collected from the electronic medical records of each participating institute. Informed consent was not obtained in writing because this was a prospective observational study without any invasive procedures, and the study protocol was approved by our institutional review board.

### 2.2. Patient Population and Center Assignment

**Figure 1:**
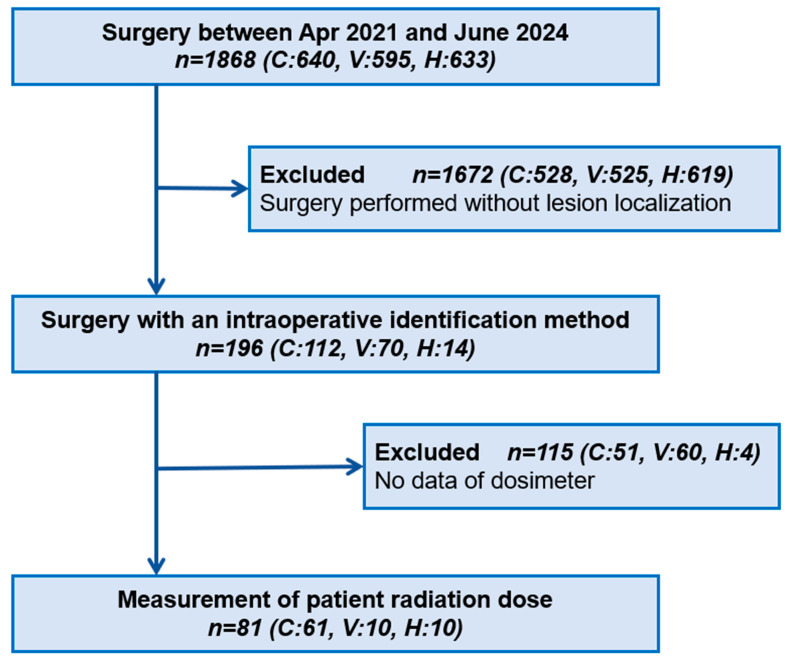
Flowchart of patient enrolment. We collected data on all surgeries requiring an intraoperative identification method at three institutions from April 2021 to June 2024. C: cone-beam computed tomography, V: virtual-assisted lung mapping, H: hook-wire.

Patients were enrolled based on institutional expertise and established clinical protocols: CBCT at Teikyo University Hospital (thoracic surgery applications in hybrid OR initiated in 2019), VAL-MAP at Shonan Kamakura General Hospital (program established 2015), and CT-guided hook-wire at Saitama Cardiovascular and Respiratory Center (established 2000). This allocation strategy ensured that patients received care from experienced teams using standardized institutional protocols. Given that surgeries for small lesions requiring marking are not consistently performed across all institutions, it was challenging to prospectively accumulate a large sample size in each group. Accordingly, the target number of cases per group was set at 10 (Figure 1).

### 2.3. Inclusion and Exclusion Criteria

This study included patients who required intraoperative localization to identify small lung nodules and for whom radiation dose measurements were available. Conversely, cases were excluded if the target lesions were expected to be easily identifiable during surgery without additional localization techniques. Further details of the inclusion and exclusion criteria are provided in Table 1.

### 2.4. Radiation Dose Measurement Methodology

Patient radiation exposure was measured using standardized wearable optically stimulated luminescence dosimeters (nanoDot^®^, Nagase Landauer, Tsukuba, Japan) composed of aluminum oxide. All dosimeters underwent phantom calibration using 80-kVp X-rays with measurement uncertainty of ±10%. The calibration was traceable to national standards and performed annually to ensure accuracy across all participating institutions.

Quality assurance protocols included: Pre-procedure dosimeter verification, standardized placement by experienced surgeons, post-procedure immediate collection and processing, and systematic documentation of all radiation sources. All dosimeters from all institutions were centralized and measured by a single expert (I.K.) to eliminate inter-observer variability and ensure measurement consistency across sites.

**Figure 2:**
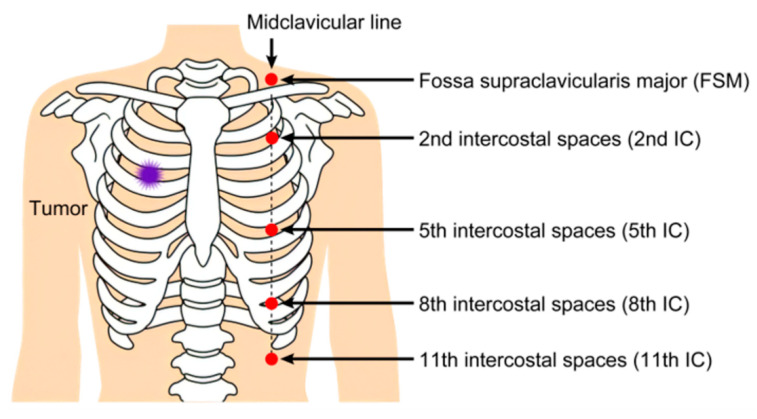
Five wearable dosimeters are placed on the patients’ bodies. These dosimeters are attached to the middle axillary line opposite the target. FSM: fossa supraclavicularis major, IC: intercostal spaces.

Five dosimeters were placed at standardized anatomical locations on the patient’s anterior chest wall, outside the sterile surgical field: fossa supraclavicularis major (FSM) and the 2nd, 5th, 8th, and 11th intercostal spaces (IC) at the midclavicular line. The total radiation dose was calculated as the sum of measurements from all five dosimeters (Figure 2).

### 2.5. Cone-Beam CT

At Teikyo University Hospital, a hybrid OR was prepared for patients with thoracic diseases requiring intraoperative localization. A CBCT (Allura Xper FD20, Philips Healthcare, Eindhoven, The Netherlands) and a free-floating table (Maquet GmbH, Rastatt, Germany) were used for the intraoperative identification of target lesions in the chest. All patients were intubated with a double-lumen endobronchial tube under general anesthesia and fitted with five dosimeters, as previously described. Subsequently, the patients were placed in the lateral decubitus position, and the initial CBCT scan was performed for skin marking. Metal clips (Ligaclip ER420, Johnson & Johnson K.K., Tokyo, Japan) were used to place markings on the visceral pleura. The second scan was performed while the lungs were held in a fully inflated state by the anesthesiologist under general anesthesia. The CBCT scans could be repeated until the precise spatial relationship between the target lesions and metal clips was completely understood. In this method, the patient’s radiation exposure doses were recorded from multiple CBCT scans in a hybrid OR.

### 2.6. VAL-MAP

VAL-MAP, a preoperative bronchoscopic multispot dye-marking technique for identifying small pulmonary nodules, was proposed in 2014 and introduced at the Shonan Kamakura General Hospital in 2015 [17,21]. Lung mapping was planned by thoracic surgeons using conventional CT imaging and virtual bronchoscopy and fluoroscopy using Ziostation (Ziosoft, Inc., Tokyo, Japan) on the day before surgery. All patients were fitted with five dosimeters as previously described and placed on an examination table in a fluoroscopy room. Bronchoscopic dye mapping was performed under fluoroscopic guidance (CUREVISTA, Hitachi, Ltd., Tokyo, Japan) using a blunt-tip catheter (P6-CW-1, Olympus Corporation, Tokyo, Japan), which was preloaded with indigo carmine. In this procedure, intravenous anesthesia combined with topical anesthesia was applied to the trachea and bronchus. After VAL-MAP, the patients underwent LDCT examination (Aquilion PRIME TSX-303A, Canon Medical Systems Corporation, Otawara, Japan) for localization of the marking sites. In VAL-MAP, patient radiation exposure was determined through fluoroscopic bronchoscopy and LDCT examination on the day before surgery.

### 2.7. Hook-Wire Technique

Perioperative percutaneous needle localization under CT guidance was proposed in 1992, and CT-guided hook-wire placement for small peripheral pulmonary nodules was introduced at the Saitama Cardiovascular and Respiratory Center in 2000 [9,10,22]. All patients initially underwent standard diagnostic chest CT with 1 or 5 mm collimation (Discovery HD750, GE Healthcare, Waukesha, WI, USA). If it was judged that the target lesion could not be identified from the pleural surface through visual inspection or palpation using an instrument, the patient was placed on the CT table in a position (supine, prone, lateral decubitus, or oblique, depending on the target lesion) that allowed the shortest possible direct access route for needle placement on the day of surgery. Before the initial chest CT scan, all patients were fitted with five dosimeters as previously described. To confirm the target location, several preliminary contiguous CT images with 1.25 mm collimation were obtained with the patient’s lungs at full inspiration. After applying local anesthesia to the skin, the Guiding-Marker System (Hawk Hook Wire, Hokkaido Medical Co., Ltd., Nagano, Japan) was advanced under CT guidance to a position near the target. Furthermore, additional CT images were obtained to confirm the route of the localization needle. The CT device used for this procedure was the same as that used for diagnostic CT. Subsequently, the hook-wire was introduced through the needle, and the needle was removed. After the procedure, several CT images were obtained to confirm the position relative to the target lesion and detect any complications, including pneumothorax, bleeding, and/or air embolism. The external suture was shortened and covered with sterile gauze, and the patients were immediately taken to the patient room. Eventually, they were transported to the OR, where the hook-wire became thoracoscopically visible under general anesthesia with one-lung ventilation. In this method, patient radiation exposure was determined through several CT examinations before and after placement of the hook-wire on the day of surgery.

### 2.8. Statistical Analysis and Power Calculation

The radiation doses measured by the five dosimeters were summed and compared across the methods. Statistical analysis included assessment of normality using Shapiro–Wilk tests and homogeneity of variances using Levene’s test. Based on assumption testing results, data transformation was performed as appropriate prior to analysis. One-way analysis of variance (ANOVA) was performed with post hoc comparisons using Tukey’s Honestly Significant Difference (HSD) test. Effect sizes were calculated using eta-squared (η^2^) and omega-squared (ω^2^) with 95% confidence intervals. To ensure robustness of parametric results, non-parametric sensitivity analysis was conducted using Kruskal–Wallis test, followed by post hoc pairwise comparisons using Mann–Whitney U tests with Bonferroni correction for multiple comparisons.

All analyses were conducted using the EZR software (Easy R ver. 4.3.1, Saitama Medical Center, Jichi Medical University, Saitama, Japan), and *p* < 0.05 was considered to indicate statistical significance.

## 3. Results

### 3.1. Patient Characteristics

**Figure 3:**
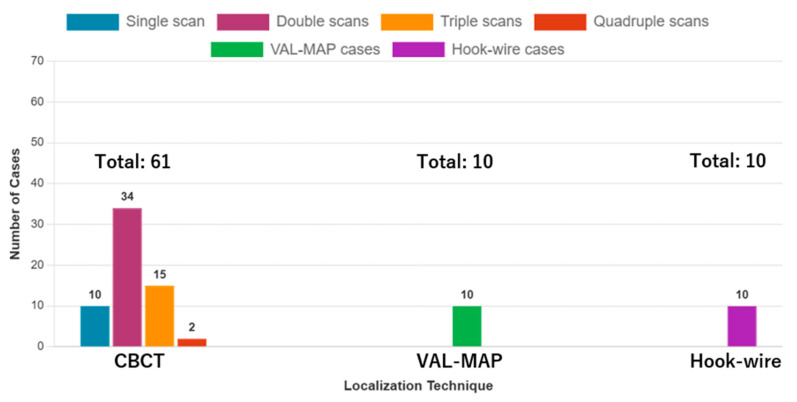
Distribution of cases by facility and CBCT scan frequency. The bar chart shows the number of cases for each localization technique (CBCT = 61, VAL-MAP = 10, Hook-wire = 10) and the distribution of CBCT cases by scan frequency: single scan (*n* = 10), double scans (*n* = 34), triple scans (*n* = 15), and quadruple scans (*n* = 2). CBCT: cone-beam computed tomography, VAL-MAP: virtual-assisted lung mapping.

The patient characteristics are summarized in Table 2. The patients’ mean age was 64.1 (±12.7) years, and 47 (57.3%) of them were men. The marking methods included CBCT (*n* = 61, 75.3%), VAL-MAP (*n* = 10, 12.3%), and hook-wire (*n* = 10, 12.3%). For CBCT, there were 10 cases with a single scan (16.4%), 34 with double scans (55.7%), 15 with triple scans (24.6%), and 2 with quadruple scans (3.3%); there was no case with more than five scans (Figure 3). There were 66 cases with a single lesion (81.5%), 10 with double lesions (12.3%), 3 with triple lesions (3.7%), and 2 with more than four lesions (2.5%). Of the two cases that required quadruple CBCT scans, one involved multiple lesions (three lesions), while the other had a solitary lesion. The surgical procedures included single-wedge resection (*n* = 62, 76.5%), bi-wedge resection (*n* = 8, 9.88%), segmentectomy (*n* = 6, 7.41%), lobectomy (*n* = 4, 4.94%), and lobectomy combined with wedge resection (*n* = 1, 1.23%). In all four cases that underwent lobectomy, an initial wedge resection was performed, and intraoperative frozen section diagnosis confirmed primary lung cancer, prompting the subsequent lobectomy. The mean values of tumor and invasive sizes preoperatively were 12.8 (±6.12) mm and 9.28 (±7.16) mm, respectively, and 12.8 (±6.75) mm and 9.54 (±7.10) mm on pathological examination. On pathological diagnosis, there were 42 cases of primary lung cancer (51.9%) and 23 cases of metastatic tumors (28.4%). All three groups achieved a lesion resection rate of 100%.

### 3.2. Statistical Assumptions and Analysis Approach

Prior to analysis, normality was assessed using Shapiro–Wilk tests for each group: VAL-MAP (W = 0.848, *p* = 0.055), hook-wire (W = 0.943, *p* = 0.585), single-scan CBCT (W = 0.938, *p* = 0.531), double-scan CBCT (W = 0.978, *p* = 0.723), and triple-scan CBCT (W = 0.867, *p* = 0.031). While most groups showed approximate normality, the triple-scan CBCT group deviated significantly from normality. Homogeneity of variances was assessed using Levene’s test, which indicated equal variances across groups (F = 0.795, *p* = 0.532). Given the mixed normality results, data were log-transformed prior to analysis.

### 3.3. Primary Endpoint: Total Radiation Dose

**Figure 4:**
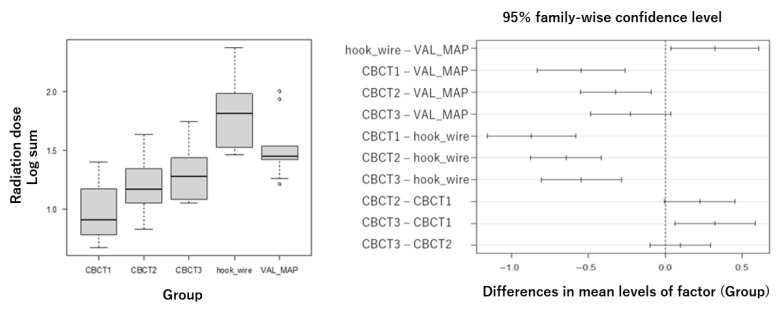
Box plot showing total radiation dose (sum of five dosimeters) with standard deviation error bars. Statistical analysis was performed using one-way analysis of variance (ANOVA) on log-transformed data, followed by Tukey’s post hoc test. The quadruple-scan CBCT group (*n* = 2) is shown for descriptive purposes only and was excluded from statistical comparisons. CBCT: cone-beam computed tomography, CBCT1: single-scan CBCT, CBCT2: double-scan CBCT, CBCT3: triple-scan CBCT, VAL-MAP: virtual-assisted lung mapping.

The primary analysis revealed highly significant differences in total radiation exposure among techniques (Figure 4). Mean total doses ± standard deviation were: Hook-wire: 86.9 ± 61.7 mGy (*n* = 10), VAL-MAP: 39.8 ± 27.5 mGy (*n* = 10), single-scan CBCT: 11.0 ± 6.5 mGy (*n* = 10), double-scan CBCT: 17.3 ± 7.8 mGy (*n* = 34), triple-scan CBCT: 23.1 ± 14.0 mGy (*n* = 15). For the quadruple-scan CBCT group (*n* = 2), descriptive values were 22.7 ± 0.1 mGy; however, this group was excluded from formal statistical comparisons due to insufficient sample size and represents exploratory data only. One-way ANOVA on log-transformed data showed significant group differences (F(4,74) = 23.17, *p* < 0.001) with large effect sizes (η^2^ = 0.56, 95% CI 0.45–0.70; ω^2^ = 0.53, 95% CI 0.42–0.68), indicating that despite modest sample sizes, the study had sufficient statistical power to detect clinically meaningful differences. Post hoc analysis using Tukey’s HSD test demonstrated significantly lower radiation exposure with all CBCT methods compared to the hook-wire technique (*p* < 0.01), and CBCT with single and double scans showed significantly lower exposure compared to VAL-MAP (*p* < 0.01). To confirm the robustness of parametric results, non-parametric sensitivity analysis using Kruskal–Wallis test also indicated significant group differences (H = 39.2, *p* < 0.01). Post hoc non-parametric pairwise comparisons (Mann–Whitney U tests with Bonferroni correction) confirmed that hook-wire had significantly higher doses compared to all CBCT methods (adjusted *p* < 0.01), and VAL-MAP showed significantly higher doses compared to single-scan CBCT and double-scan CBCT (adjusted *p* < 0.01).

Notably, 96.7% of CBCT procedures (59/61 cases) were completed within triple scans, indicating that quadruple scans represent exceptional rather than routine cases.

### 3.4. Secondary Analysis: Anatomical Dose Distribution

Anatomical site analysis revealed consistent patterns across techniques (Table 3). Radiation dose distribution varied across anatomical sites and techniques, with the hook-wire method showing the highest doses, particularly at the 5th and 8th intercostal spaces (20.1 ± 18.5 mGy and 25.1 ± 41.4 mGy, respectively). CBCT demonstrated lower and more uniform exposure patterns across all anatomical sites, while VAL-MAP showed intermediate values with modest variability. Among all sites, the 11th intercostal space generally demonstrated the lowest exposures across all techniques.

## 4. Discussion

### 4.1. Comparison with Previous Literature

Our findings demonstrate significantly lower radiation exposure with CBCT compared to hook-wire and VAL-MAP techniques, contrary to initial expectations. Wei et al. (2023) reported hook-wire localization doses with dose-length product values ranging from 56.86 ± 4.73 to 746.01 ± 230.91 mGy·cm depending on CT protocol and patient body mass index (BMI) [23]. While direct comparison is challenging due to different measurement methodologies, our surface dose measurements of 86.9 ± 61.7 mGy align with their findings of substantial radiation exposure during multiple CT acquisitions required for wire placement confirmation.

Previous reports in other interventional fields have already examined the increase in radiation exposure with CBCT according to the number of scans, and BMI has also been identified as an important contributing factor [24,25]. The absence of BMI evaluation in the present study should be considered in future investigations. At the same time, advances in CBCT technology have improved image quality while reducing radiation exposure compared to earlier systems [26,27]. Our results extend these findings to thoracic surgery applications.

The VAL-MAP technique, while showing intermediate radiation exposure in our study, has limited comparative data in the literature [28]. Sato et al.’s original series focused primarily on technical feasibility and safety outcomes rather than detailed dosimetry [17,18]. Our findings provide the first quantitative radiation exposure data for this technique, revealing significant variability (39.8 ± 27.5 mGy) that may reflect procedural complexity and operator experience differences.

### 4.2. Clinical Implications and Practice Impact

The demonstration that CBCT with up to triple scans results in lower radiation exposure than alternative methods has important clinical implications. The large effect size observed in our study (η^2^ = 0.56) indicates not only statistical significance but substantial practical significance, suggesting that the radiation dose differences between techniques represent clinically meaningful variations that could influence patient safety considerations and technique selection. This finding supports the broader adoption of CBCT-guided localization, particularly for complex cases involving multiple lesions where traditional techniques would require multiple separate procedures, each carrying additional radiation exposure and complication risks. The magnitude of the effect size reinforces the robustness of our findings despite the relatively modest sample sizes, providing confidence in the clinical applicability of these results.

For institutions considering the implementation of hybrid OR technology, our data provide evidence supporting the radiation safety of CBCT-guided approaches. The ability to complete marking and resection in a single procedure under general anesthesia eliminates risks of marker migration and patient discomfort associated with awake procedures.

In this study, two cases required quadruple scans. However, due to the limited number of such cases, it is difficult to draw definitive conclusions regarding whether CBCT consistently results in lower radiation exposure compared to other methods in these situations. Although the total radiation dose in the two cases with quadruple scans was lower than that in the triple-scan group, this is likely due to coincidental factors. Intraoperative fluoroscopy is occasionally used for minor positional adjustments in addition to CBCT scans, and it is presumed that the two quadruple-scan cases happened to require less additional fluoroscopy exposure. Notably, only one of the two cases involved three lesions. Accurate lesion identification with CBCT requires adequate lung inflation at the time of imaging. If atelectasis remains, repeat imaging may be necessary. Thus, a thorough understanding of the technique by anesthesiologists is essential. In four out of five cases (80%) with three or more lesions, scanning was completed within three sessions, indicating that CBCT is also useful in surgeries targeting multiple small lesions. In the other methods compared in this study, all cases involved two or fewer target lesions. When targeting three or more lesions, not only increased radiation exposure but also a higher risk of complications can be anticipated. A notable advantage of CBCT is that, even when the number of scans increases, it poses no additional risk apart from radiation exposure.

### 4.3. Factors Influencing Radiation Dose Variation

Several factors contribute to observed dose variations within each technique:

**Operator Experience**: Procedural duration and radiation exposure correlate with operator familiarity. Experienced operators typically require fewer repeat scans and more efficient fluoroscopy utilization.
**Patient Factors**: Body habitus affects radiation attenuation and required exposure parameters. Larger patients generally require higher radiation doses for adequate image quality.
**Lesion Characteristics**: Deeper or smaller lesions may require additional imaging for precise localization.
**Technical Parameters**: Equipment age, calibration status, and institutional protocols significantly influence radiation output. Standardization of imaging parameters and regular equipment maintenance are essential for dose optimization. To reduce the number of CBCT imaging, it is necessary to eliminate failed imaging caused by unilateral ventilation due to insufficient communication between the anesthesiologists and radiologic technologists.

### 4.4. Study Strengths and Limitations

This study represents the first direct comparison of radiation doses across these three localization techniques using standardized measurement methodology. The multicenter design enhances generalizability, while prospective data collection ensures complete and accurate dosimetry information. With the recent development of increasingly precise localization techniques [29,30], our study provides fundamental baseline data on radiation exposure.

However, several limitations must be acknowledged. First, each technique was performed at a single institution, potentially introducing institutional bias regarding protocols, equipment, and operator experience. Standardized dosimetry methods and similar patient populations mitigate this concern; however, further case registrations from a multi-institutional study with various protocols are expected to improve bias. Second, the relatively small sample sizes, particularly for hook-wire and VAL-MAP groups (*n* = 10 each), reflect the specialized nature of these procedures and limited case volumes at individual centers. Larger multicenter studies are needed to confirm our findings. Third, our measurements represent surface doses rather than effective whole-body doses. While this approach enables direct comparison across techniques, it does not provide organ-specific risk estimates. Fourth, different institutions used different equipment models, potentially affecting radiation output characteristics. However, all systems met current safety standards, and dosimeter calibration was standardized. Fifth, patient assignment to techniques was based on institutional expertise rather than randomization, potentially introducing selection bias. Nevertheless, patient characteristics were well-matched across groups. Sixth, this study focused on immediate radiation exposure without long-term clinical outcome assessment, limiting our ability to evaluate the relationship between localization techniques and surgical efficacy or patient prognosis.

Despite these limitations, our findings provide valuable baseline comparative data that were previously unavailable in the literature. Future research should address these constraints through large-scale randomized multicenter trials that implement multiple localization techniques at each participating institution, incorporate effective dose calculations, and include long-term follow-up for surgical outcomes and oncological results. Such studies would strengthen the evidence base for optimal localization technique selection and inform evidence-based clinical practice guidelines.

## 5. Conclusions

This multicenter prospective comparative study demonstrates that intraoperative CBCT localization, when limited to triple scans or fewer, results in significantly lower patient radiation exposure compared to CT-guided hook-wire placement and VAL-MAP techniques. CBCT also proved feasible for complex cases involving multiple lung lesions, where traditional methods would require multiple separate procedures with cumulative radiation exposure.

These findings have immediate clinical implications for the appropriate selection of localization techniques, particularly in institutions with hybrid OR capabilities. The lower radiation exposure profile of CBCT, combined with its safety advantages and procedural efficiency, supports its adoption as a preferred method for small lung lesion localization. Future research should focus on refining localization techniques and evaluating their broader applicability.

## Figures and Tables

**Table 1:** Structured inclusion and exclusion criteria.

Criteria	Details
***Inclusion Criteria***	
Age	≥18 years
Lesion characteristics	Small lung nodules (≤30 mm diameter) requiring intraoperative localization
Imaging	Lesions not easily identifiable on visual inspection or instrument palpation
Dosimetry	Complete radiation dose measurements available
Surgical candidacy	Suitable for minimally invasive thoracic surgery
***Exclusion Criteria***	
Lesion visibility	Target lesions easily identifiable without additional localization
Pregnancy	Current pregnancy or possibility thereof
Technical factors	Dosimeter placement failure or malfunction
Medical contraindications	Severe comorbidities precluding safe anesthesia

**Table 2:** Clinical characteristics of study patients (*n* = 81). Continuous variables are presented as mean ± standard deviation. CBCT: cone-beam computed tomography, VAL-MAP: virtual-assisted lung mapping.

		CBCT	VAL-MAP	Hook-Wire	*p* Value
Number		61	10	10	
Age	(years)	62.6 ± 13.36	70.9 ± 6.82	66.3 ± 11.10	0.137
Sex	Male	40	5	2	0.027
	Female	22	5	8	
Tumor total size	(mm)	10.4 ± 1.9	10.8 ± 2.3	11.6 ± 1.6	0.139
Lesions	Single	49	10	7	0.417
	Double	7	0	3	
	Triple	3	0	0	
	Multiple	2	0	0	
Location	Upper lobe	28	6	5	0.703
	Middle lobe	3	1	0	
	Lower lobe	30	3	5	
Procedure	Wedge	46	10	6	0.678
	Bi-wedge	6	0	2	
	Segmentectomy	5	0	1	
	Lobectomy	3	0	1	
	Lobetomy + wedge	1	0	0	
Diagnosis	Lung cancer	29	5	8	0.114
	Metastatic tumor	20	3	0	
	Benign tumor	3	1	1	
	Infection	3	0	0	
	Fibrosis	1	0	1	
	Other	5	1	0	
Lesion resection rate	(%) (n/N)	100 (81/81)	100 (10/10)	100 (13/13)	1.0

**Table 3:** Radiation exposure dose with five wearable dosimeters. Values are presented as mean ± standard deviation in mGy.

Site	CBCT1 (*n* = 10)	CBCT2 (*n* = 34)	CBCT3 (*n* = 15)	VAL-MAP (*n* = 10)	Hook-Wire (*n* = 10)	*p* Value *
FSM	1.50 ± 2.14	1.24 ± 1.06	1.23 ± 1.08	8.65 ± 6.50	7.76 ± 7.19	<0.001
2nd IC	2.20 ± 2.66	3.25 ± 2.75	2.85 ± 2.03	10.2 ± 7.45	19.0 ± 17.2	<0.001
5th IC	4.04 ± 2.45	6.67 ± 2.99	9.92 ± 6.02	10.6 ± 10.3	20.1 ± 18.5	<0.001
8th IC	2.61 ± 2.60	4.65 ± 4.85	7.65 ± 7.77	6.32 ± 5.99	25.1 ± 41.4	0.020
11th IC	0.67 ± 1.01	1.50 ± 3.39	1.45 ± 1.23	3.63 ± 2.03	14.5 ± 23.7	<0.001

* Tukey’s honestly significant difference test among three methods. CBCT: cone-beam computed tomography, CBCT1: single-scan CBCT, CBCT2: double-scan CBCT, CBCT3: triple-scan CBCT, FSM: fossa supraclavicularis major, IC: intercostal spaces, VAL-MAP: virtual-assisted lung mapping.

## Data Availability

The data presented in this study are available on reasonable request from the corresponding author. The data are not publicly available due to institutional policy.

## Abbreviations

The following abbreviations are used in this manuscript:

LDCT	Low-dose computed tomography
CT	Computed tomography
VAL-MAP	Virtual-assisted lung mapping
CBCT	Cone-beam computed tomography
OR	Operating room
FSM	Fossa supraclavicularis major
IC	Intercostal
ANOVA	Analysis of variance
HSD	Honestly Significant Difference
BMI	Body mass index
